# Differentiating pelvic actinomycosis from advanced ovarian cancer: a report of two cases, management reflections and literature review

**DOI:** 10.1186/2053-6844-1-5

**Published:** 2014-12-10

**Authors:** Alex Laios, Iryna Terekh, Hooman Soleymani Majd, Pubudu Pathiraja, Sanjiv Manek, Krishnayan Haldar

**Affiliations:** Gynaecologic Oncology Unit, Churchill Hospital, Oxford University Hospitals, NHS Trust, Oxford, UK; Department of Cellular Pathology, Oxford University Hospitals, Oxford, UK

## Abstract

Pelvic actinomycosis comprises a rare, subacute to chronic bacterial infection characterised by suppurative and granulomatous inflammation. Diagnosis is difficult as it may simulate pelvic malignancies. Laboratory and radiological findings are non-specific. We reported on 2 cases of pelvic actinomycosis mimicking ovarian malignancy with different management approaches that lead to opposite outcomes. We reviewed the literature on pelvic actinomycosis imitating ovarian cancer with a focus on its surgical management. Despite agreement on the duration of antibiotic therapy following surgical management, consensus regarding surgical approach was rather equivocal. We concluded that pelvic actinomycosis should be strongly suspected in women with presumed ovarian cancer of atypical presentation and a history of intrauterine devices (IUD).

**Electronic supplementary material:**

The online version of this article (doi:10.1186/2053-6844-1-5) contains supplementary material, which is available to authorized users.

## Background

Actinomycosis comprises a subacute to chronic bacterial infection caused by filamentous, gram-positive, nonacid-fast, anaerobic bacteria. It is characterised by contiguous, suppurative and granulomatous inflammation. Pelvic actinomycosis although rare, occurs almost only in women. It may simulate pelvic malignancies or retroperitoneal tumours [1], which often makes it difficult to diagnose. Pelvic organs can be affected leading to different clinical presentations. A high index of disease suspicion in patients with a history of intrauterine devices (IUD) can prevent unnecessary extensive surgical procedures [2]. Radiological findings are non-specific, however computed tomography (CT) appears to be the most useful imaging modality. We presented 2 case reports of pelvic actinomycosis with different management approaches that lead to opposite outcomes. We also conducted a literature review to add on the understanding of this rare disease.

## Case presentation

### Case report 1

A 57-year old, mother of two, postmenopausal caucasian woman was referred to our centre following MDT discussion for a 4-month long, persistent, progressively worsening lower abdominal pain. This was associated with mild, offensive vaginal discharge but no vaginal bleeding. She had a significant weight loss (19 kg over 1 month), loss of appetite, dysuria and constipation. Her past medical history included bipolar disorder, hypothyroidism and hypertension. She had a recent psychotic episode following discontinuation of her medication due to lithium toxicity. She had no previous abdominal or pelvic surgeries, abdominal wall trauma or immunosuppression. The patient was previously on HRT for 8 years and had a copper IUD *in situ* for the last 9 years. Her family history was unremarkable.

**Figure 1:**
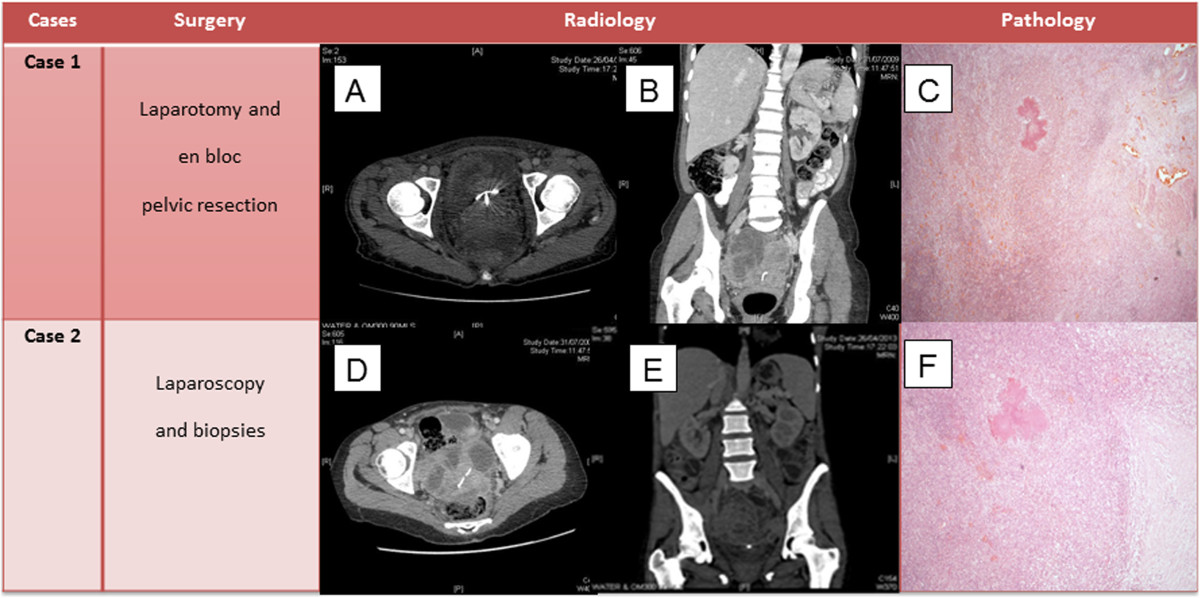
**Surgical procedures, imaging and pathological findings.** Case 1 - **a)** Axial preoperative abdominopelvic CT scan showing inseparable bilateral ovarian masses with coil in situ **b)** coronal view **c)** colony of Actinomyces organisms surrounded by inflammatory cells with adjacent fibrosis which had disrupted the smooth muscle of bowel wall rendering a ‘mass’ (H&E stain, 10×). Case 2 **- d)** preoperative abdominopelvic computed tomography (CT) scan showing right complex adnexal mass (axial) **e)** preoperative abdominopelvic CT showing presacral mass with IUD in situ (coronal) **f)** colony of Actinomyces surrounded by acute and chronic inflammatory cells. To the right of the image, there is fibrosis (H&E stain, 10×).

**Figure 2:**
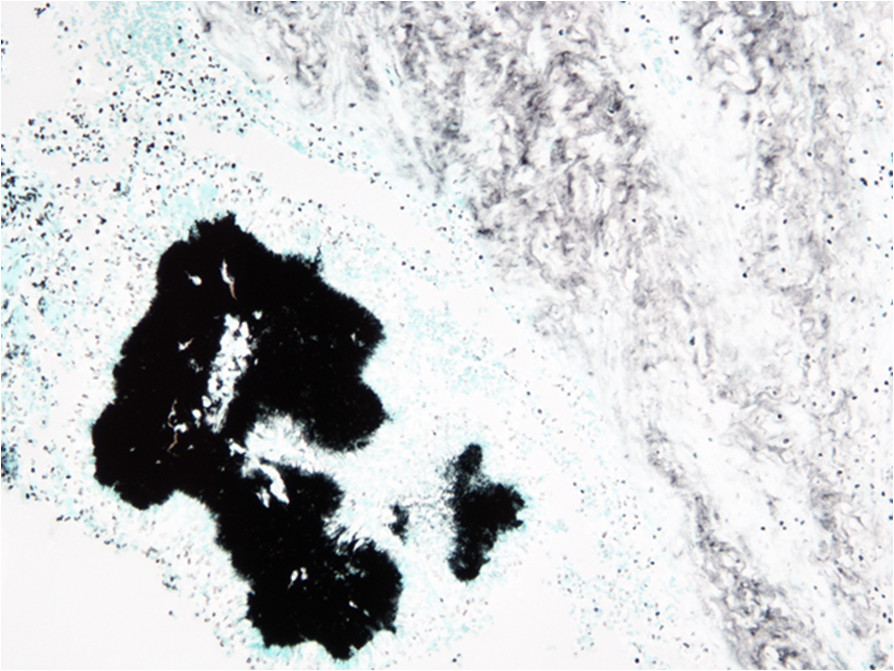
**Colony of Actinomyces organisms (Grocott stain, 40×)**.

**Figure 3:**
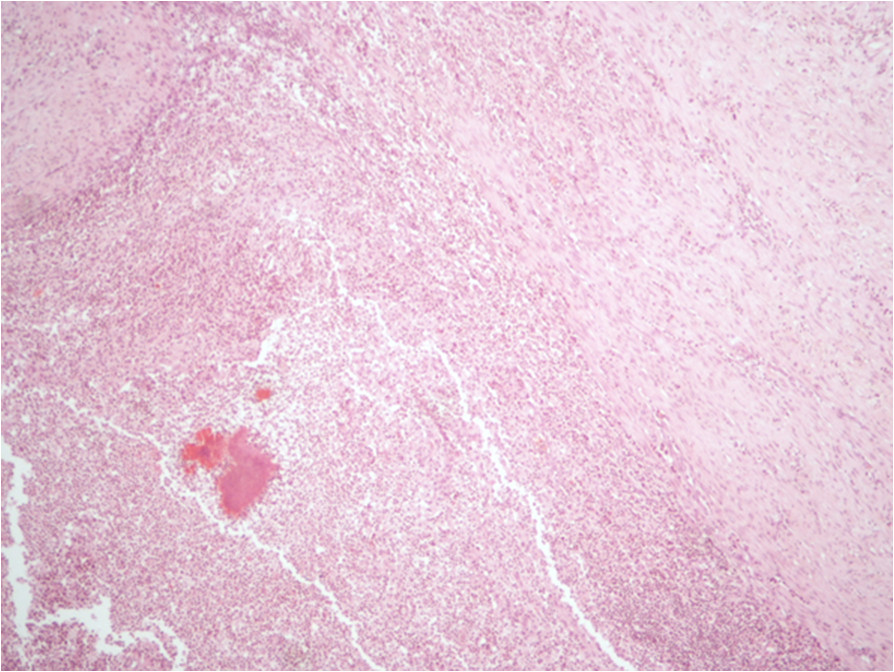
**Mass effect.** Another area in the exenteration sample in case 1 showing the inflammation and fibrosis which has caused a mass effect (H&E stain, 10×).

Physical examination demonstrated a large abdominal mass protruding through the anterior abdominal wall between the umbilicus and symphysis pubis. She was afebrile and had mild generalised abdominal tenderness but no rigidity or guarding. Pelvic examination revealed a 20-week sized gravid uterus and a fixed solid abdominopelvic mass with no cervical motion tenderness. Laboratory investigations demonstrated mild anaemia (Hb: 9 g/dL), raised CRP at 118 mg/l and leucocytosis (WCC 27 × 10^3^/μl).A pelvic US showed bilateral complex, predominantly cystic pelvic masses, inseparable from each other. This was confirmed by CT, which additionally reported a soft anterior abdominal wall mass 60 × 25 × 60 mm situated below the umbilicus involving the right rectus abdominis muscle. A small amount of fluid in the right paracolic gutter was seen in addition to generalised peritoneal disease and sigmoid involvement. The overall picture was suggestive of malignant ovarian disease (Figure 1a-b).CA125 was 39 U/ml, CEA and CA199 were normal. The patient was transfused preoperatively due to low haemoglobin and scheduled for an exploratory laparotomy preceded by laparoscopy. Unfortunately, due to deterioration of her symptoms, she underwent an emergency procedure. Following initial laparoscopic assessment to assess the disease operability, she underwent an en bloc pelvic resection with total abdominal hysterectomy and bilateral salpingo-ophorectomy, omentectomy, bladder peritonectomy, rectosigmoid resection with re-anastomosis and excision of an anterior abdominal wall tumour. Subsequently, she required a defunctioning stoma due to pelvic sepsis and partial closure of the abdominal defect with a prolene mesh. Further debridement was required, which was followed by a split skin graft from her right thigh. Histology revealed widespread abdominal and pelvic actinomycosis with a florid inflammatory and fibrotic response comprising of microabscesses (Figures 1c and 2). A tumour mass effect was seen (Figure 3). Active chronic inflammation in the endometrial cavity, most likely associated with the IUD was reported. She remained on IV benzylpenicillin 1.8 mg/4 h for a total of 6 weeks. She was discharged at 2 months with a low grade wound infection secondary to foreign mesh material reaction and a rather erratic colostomy performance required supportive management. She became systemically well. Oral amoxicillin 500 mg 3 times daily was initially considered until reversal of the colostomy. At 9 months follow-up, endoscopy showed mild active proctitis and as it deemed unsafe to restore GI continuity, a revision of her end colostomy with reconstruction of abdominal wall was undertaken. The total duration of oral antibiotics was 12 months.

### Case report 2

A 37-year old, mother of two, caucasian woman was initially presented to the gastroenterology team for investigation of a 3-month long persistent, progressively worsening lower abdominal pain. This was severe in nature, predominant in the left flank, lasting for a few seconds and spontaneously resolving. It was associated with significant weight loss (15 kg over 2 months), pyrexia, night sweats, anorexia and altered bowel habits. Her medical history included asthma. She had no previous abdominal surgery, trauma or immunosuppression. She had a copper IUD *in situ* for 4 years. There was a family history of colon cancer and Crohn’s disease.

On physical examination, there was no pyrexia. There was left flank and iliac fossa tenderness with no rigidity or guarding and mild left lower limb oedema. Pelvic examination revealed a tender, solid, fixed pelvic mass extending to the left pelvic side wall and the posterior sacral region. Doppler ultrasonography (US) of the left lower limb ruled out deep vein thrombosis and superficial thrombophlebitis. Laboratory investigations demonstrated anaemia (Hb: 7.9 g/dL), raised CRP at 133 mg/l and leucocytosis (WCC 14.3 × 10^3^/μl).Gastro-duodenoscopy revealed mild chronic gastritis and reactive gastropathy whilst colonoscopy ruled out the possibility of inflammatory bowel disease. CT thorax-abdomen-pelvis scan revealed a 75 × 61 mm complex right adnexal mass and a 52 × 41 mm heterogeneous pre-sacral mass. Multiple smaller masses were seen in the left flank, left iliac fossa and anterior pelvis, consistent with metastatic disease (Figure 1d-e). Several loops of small bowel appeared to be tethered in the pelvis and were partially obstructed. The rectum was thought to be infiltrated by the pre-sacral mass. There were bilateral hydro-nephroses. There was small volume of ascites with some associated peritoneal enhancement and mild left pleural effusion with no evidence of lung parenchymal metastases.

The case was discussed at the gynaecologic oncology multidisciplinary team (MDT) due to high suspicion of advanced ovarian cancer. Tumour markers included elevated CA125 at 83 U/ml, CA199 at 51 U/ml; AFP and CEA were normal. A US-guided percutaneous biopsy of the left adnexal mass was performed as per MDT decision, which was abandoned as there was no safe path to the lesion and hence diagnostic laparoscopy and biopsies were planned instead.At laparoscopy, there were multiple omental and large bowel adhesions to the anterior and lateral abdominal wall. The right adnexal mass was not visualised as the adjacent small bowel was obscuring the right ovary. A left pelvic side wall biopsy was taken and ascitic fluid was drained. Histology confirmed a benign peritoneal nodule showing fibrosing inflammation in association with actinomyces colonies. The latter were surrounded by acute suppurative exudate within chronic inflammation and prominent foamy macrophages (Figure 1f). The fibrous band rendered a mass effect. No dysplasia or malignancy was present. Cytology was negative for malignancy too. This was therefore a case of severe pelvic inflammatory disease secondary to actinomyces infection. The patient made an uneventful recovery. She had a negative STD work-up and the IUD was removed. However, IUD culture failed to isolate actinomyces. Blood cultures were negative for bacteraemia. She was advised against reinsertion of the IUD.

The patient was commenced on intravenous (IV) benzylpenicilline 1.8 mg 4-hourly followed by IV ceftriaxone 2 g daily for 6 weeks. She was then switched to oral amoxicillin 500 mg 3 times daily totalling 6 months. As a result, her weight increased steadily whilst her night sweats and abdominal discomfort subsided. All haematological parameters returned to normal. Abdominopelvic examination prior to discharge was unremarkable. She returned to follow-up appointments at 6 weeks and 6 months and was completely asymptomatic.

### Literature review

We searched the MEDLINE and EMBASE databases for articles published between 1988 and 2013 using medical subject heading (MeSH) terms. Key terms included “pelvic actinomycosis” and “ovar* cancer or tumour or carcinoma or neoplasm”. The search was limited to the words “humans and adult female”. Additional publications were identified via cross-referencing from reference lists within the retrieved publications. Only case series published in English language but with no geographical restrictions were included in the literature review.

## Results

**Figure 4:**
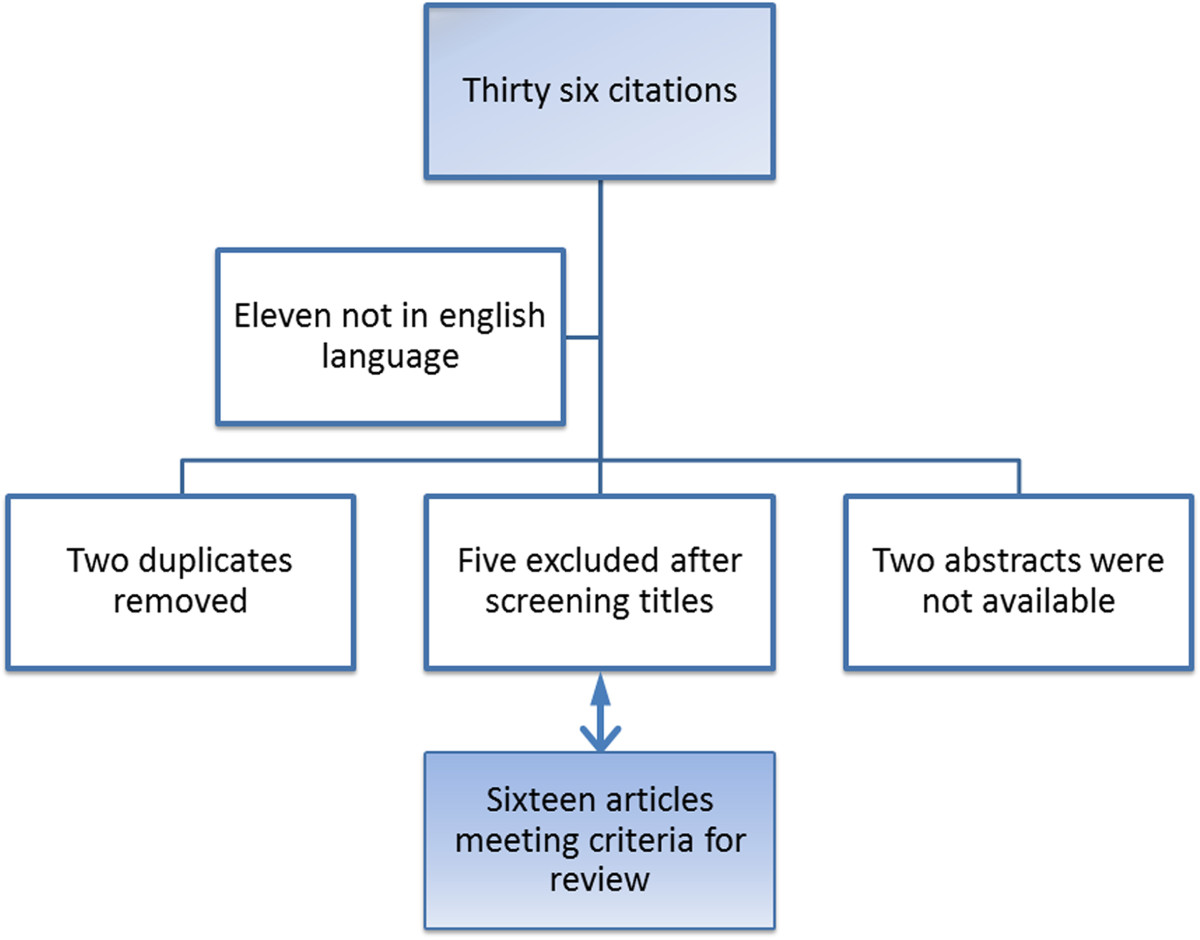
**Comprehensive review selection strategy.**

The electronic search initially yielded 36 citations. Eleven reports were published in language other than english. There were two duplicate studies. Five studies were unrelated after screening titles; one study was a review, 3 studies referred to bladder cancer, intrauterine myomas and tubo-ovarian abscesses respectively and one study was on a pediatric patient. In 2 studies, abstracts were not available and they were further excluded. A total of 16 publications were finally included in the literature review (Figure 4). The main characteristics of those case report studies with an emphasis to their management are shown in Table 1.

**Table 1:** **Case report studies on pelvic actinomycosis mimicking advanced ovarian cancer**

Case report studies on pelvic actinomycosis mimicking advanced ovarian cancer				
Reference	Age (years)	Title	Duration of copper IUD (years)	Diagnosis/Management algorithm/complications
Gungor T et al. [3], 2013 (n = 1)	43	Pelvic actinomycosis: a disease that should not be overlooked in cases with suspected ovarian cancer	7	Explorative laparotomy and debulking surgery
Kim YS et al. [4], 2012 (n = 1)	41	Metastatic hepatic actinomycosis masquerading as distant metastases of ovarian cancer	15	Hepatic actinomycosis misdiagnosed as distant metastases of ovarian cancer, exploratory laparotomy with frozen section of pelvic mass, penicillin totalling 5 months
Ong C et al. [5], 2012 (n = 1)	73	Actinomyces turicensis infection mimicking ovarian tumour	No IUD	Pelvic mass and enlarged supraclavicular LN, non-diagnostic FNA, blood cultures positive for actinomyces, penicillin totalling 7 months
Pusiol T et al. [2], 2011 (n = 1)	46	Abdomino-pelvic actinomycosis mimicking malignant neoplasm	3	Uncomplicated explorative laparotomy and debulking surgery but incomplete staging, penicillin totalling 6 months
Hwang JH et al. [6], 2010 (n = 1)	59	Primary serous papillary carcinoma of the peritoneum mimicking pelvic actinomycosis: a case report and brief literature review	No IUD	Pelvic CT and MRI suggestive of pelvic actinomycosis. Full staging debulking surgery confirmed primary peritoneal carcinoma followed by adjuvant chemotherapy
Kumar N et al. [7], 2010 (n = 1)	32	Pelvic actinomycosis mimicking an advanced ovarian cancer	2	IUD removed 4 months prior to admission, uncomplicated explorative laparotomy and debulking surgery, incomplete staging
Lee YK et al. [8], 2008 (n = 1)	42	Pelvic actinomycosis with hydronephrosis and colon stricture simulating an advanced ovarian cancer	8	Imaging guided needle biopsy made correct nonsurgical diagnosis, antibiotic treatment, no surgery required
Akhan SE et al. [9], 2008 (n = 3)	38, 37, 51	Pelvic actinomycosis mimicking ovarian malignancy: three cases	>7	Debulking surgeries in all cases, complete/incomplete staging, second case was complicated by need for colostomy and fascial necrosis
Kim HS et al. [10], 2007 (n = 1)	47	A case of pelvic actinomycosis with hepatic actinomycotic pseudotumor	18	FNA of hepatic tumour and explorative laparotomy of pelvis masses followed by penicillin totalling 4 months
Sehouli J et al. [11], 2006 (n = 1)	35	Actinomycotic inflammatory disease and misdiagnosis of ovarian cancer	10	Ureteric stenting followed by uncomplicated explorative laparotomy, patient received ampicillin and sulbactam totalling 6 weeks
Atay Y et al. [12], 2005 (n = 3)	37, 45, 47	Ovarian actinomycosis mimicking malignancy	No IUD	Explorative laparotomy and intraoperative frozen section diagnosis of actinomycosis, long-term penicillin totalling 3 months
Oztekin K et al. [13], 2004 (n = 1)	49	Pelvic actinomycosis in a postmenopausal patient with systemic lupus erythematosus mimicking ovarian malignancy; case report and review of literature	No IUD	Rare occurrence of actinomycosis with an autoimmune disease that predisposed to infections, patient had laparotomy
Koshiyama M et al. [14], 1999 (n = 1)	52	Ovarian actinomycosis complicated by diabetes mellitus simulating an advanced ovarian carcinoma	No IUD	MRI detected solid pelvic tumour mimicking advanced ovarian carcinoma invasive to bladder, rectum and uterus, patient denied initial explorative laparotomy, had neodjuvant chemotherapy followed by incomplete staging laparotomy and long-term penicillin, colostomy secondary to rectovaginal fistula
Hawnaur JM et al. [15], 1999 (n = 1)	43	Magnetic resonance imaging of actinomycosis presenting as pelvic malignancy	10	MRI confirmed regression of pelvic disease in response to antibiotic therapy
Kirova YM et al. [16], 1997 (n = 1)	37	IUD-associated pelvic actinomycosis: a rare disease mimicking advanced ovarian cancer; a case report	4	CT detected pelvic mass with liver metastases mimicking advanced ovarian carcinoma, exploratory laparotomy and debulking surgery, incomplete staging
Hoffman MS et al., [17], 1991 (n = 2)	N/A	Advanced actinomycotic pelvic inflammatory disease simulating gynecologic malignancy. A report of two cases.	Plastic IUCD, 7, 17	Laparotomy and long-term penicillin, some resolution of the pelvic fibrosis

## Discussion

**Figure 5:**
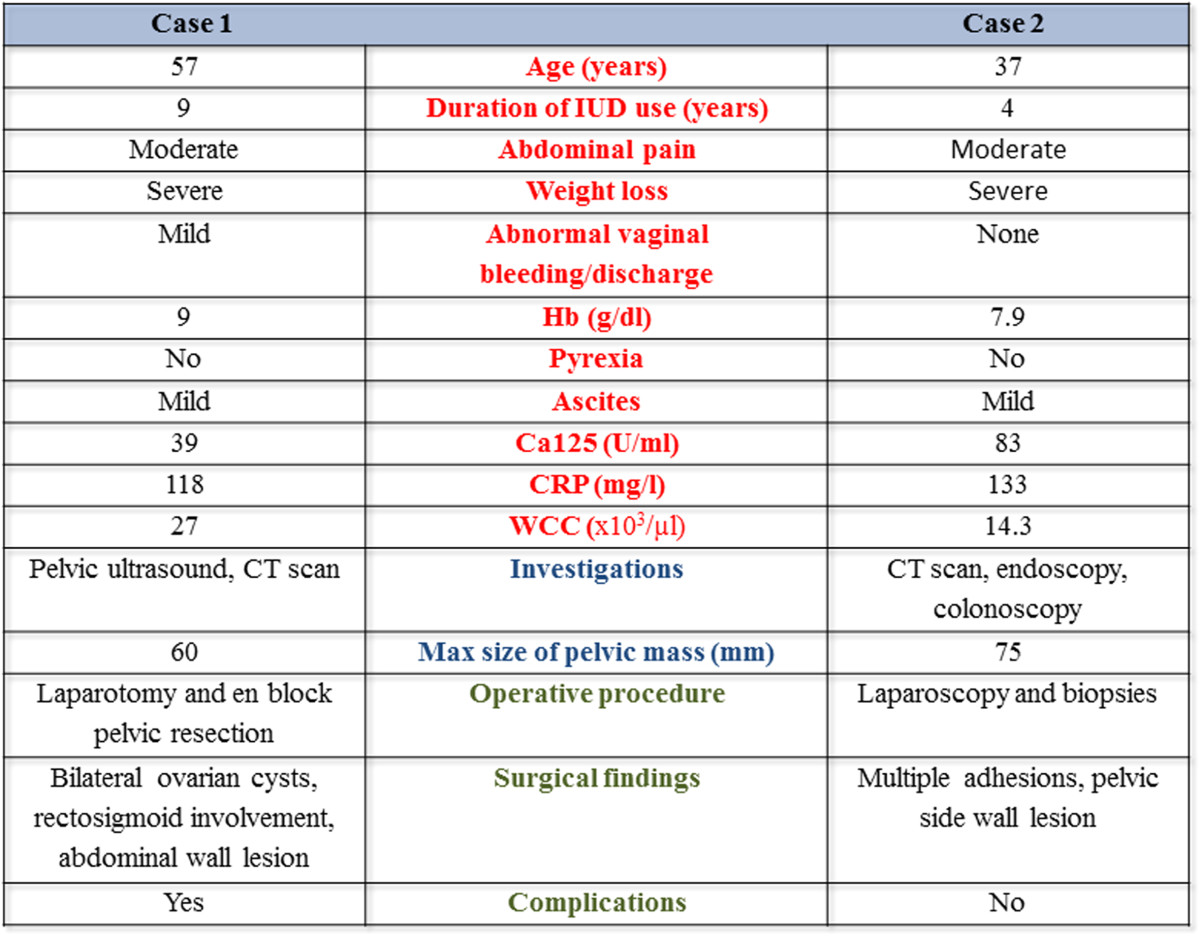
**Differentiation between the two reported cases with pelvic actinomycosis.**

Our institution is a tertiary referral cancer centre; as large numbers of patients are referred with presumed metastatic ovarian or primary peritoneal cancer, it is important to determine the frequency and nature of diseases that mimic malignancy. In this context, pelvic actinomycosis, although difficult to diagnose by virtue of its rarity [18], should be included in the differential diagnosis of ovarian cancer, especially if atypical presentation occurs.We reported 2 cases of women who were diagnosed with extensive pelvic actinomycosis following surgical intervention. All management decisions were MDT approved and documented in MDT proformas. Their different management demonstrates the importance of making the diagnosis in an evocative clinical context, performing the necessary investigations to confirm suspicions, favouring medical treatment and possibly reserving surgical treatment for specific situations (Figure 5).

Both patients had a long standing history of copper IUD use. Pelvic actinomycosis accompanied by IUD accounts for about 3% of all actinomycosis [19]. Although uncommon, a long duration of IUD appears to confer the greatest risk [1]. Signs of infection such as fever, night sweats and leucocytosis also add suspicion towards an infective cause or accurately predict the severity of the condition [20]. In our cases, abdominopelvic pain associated with significant weight loss constituted the prominent symptoms. As ovarian malignancy was presumed in both cases, staging CT scans were included in the diagnostic work-up. CT findings although useful in the preoperative diagnosis were non-specific to differentiate between malignancy and actinomycocis. They are quite often, similar to those in Crohn’s disease or intestinal tuberculosis. However, they might show the aggressive, infiltrative nature of the disease with disruption of tissue planes and demonstrate one or more solid masses with thickened walls [21]. The first patient was initially referred from a regional hospital for MDT discussion. In the light of suspected advanced ovarian cancer and while waiting for an elective procedure with the intention-to-treat, she deteriorated and transferred to our centre. She underwent an emergency laparoscopy proceeding to exploratory laparotomy with en bloc resection. In the absence of emergency, a more conservative surgical approach should have been pursued. Performing such an extensive operation in such an infected surgical field added to the complications encountered in the postoperative period. The learning experience from that first case was reflected on the MDT discussion of the second case, yet ovarian malignant disease was the working diagnosis. A biopsy taken at the time of a diagnostic laparoscopy confirmed actinomycosis infection [22]. Frozen section should be considered in cases of atypical adnexal masses before undertaking extensive surgery [23]. Recently, a high detection rate of actinomyces by cytology has been suggested adding on the diagnosis [24].

Several case reports of pelvic actinomycosis mimicking ovarian malignancies have been published [3, 25–27] and (Table 1). Pelvic actinomycosis has been known to present as a rectal mass with hydronephrosis [28], following hysteroscopic removal of IUD [29] or incarcerated inguinal hernia [30]. In the presence of an abdominopelvic mass with suspected deposits, laparoscopy was a less invasive approach of establishing a definitive diagnosis, thereby minimising the risk for mutilating surgery. In most reports pelvic actinomycosis was diagnosed in women younger than 50 years old, who would not benefit from extensive surgery, especially as medical treatment was likely to be successful. Adjuvant debulking surgery could have taken place later if medical treatment was unsuccessful or if the patient developed complications [31]. Nonetheless, Nagler suggested that patients may not respond well to antibiotics before lesion resection, possibly due to compartmentalisation of organisms within granulation tissue [32]. Aggressive surgical management including extensive surgery –full debulking- is overwhelmingly supported in the literature [3, 9, 11, 14, 33, 34] taking into consideration the extent of the disease and patient’s condition (Table 1). Alternatively, incomplete staging may be offered [2, 7, 16, 17] as an intermediate approach with lower morbidity risk while histology is pending.

According to this review, there was no consensus with respect to surgical management of actinomycosis. Agreement came only with the approximate duration of antibiotic treatment. Once diagnosis was made, high-dose and long-term use of penicillin was recommended to eradicate actinomycosis. IV benzylpenicillin was administered daily for up to 6 weeks following surgery. Oral treatment should be continued for a period of at least 6 months due to low penetration in the fibrosis and the tendency to recur [4].

## Conclusion

Despite advanced imaging and diagnostics, it is important to suspect actinomycosis in women who present with a presumed ovarian cancer and a history of IUD. This will spare patients from unnecessary, potentially extensive surgery.

## Consent

Written informed consents were obtained from the patients for publication of their case report and accompanying images.

## Authors’ original submitted files for images

Below are the links to the authors’ original submitted files for images. Authors’ original file for figure 1 Authors’ original file for figure 2 Authors’ original file for figure 3 Authors’ original file for figure 4 Authors’ original file for figure 5
