# Supplementary figures and images for: Differentiating pelvic actinomycosis from advanced ovarian cancer: a report of two cases, management reflections and literature review
Source: Gynecol Oncol Res Pract. 2014 Dec 10;1:5. doi: 10.1186/2053-6844-1-5 (PMC4877746; doi:10.1186/2053-6844-1-5)

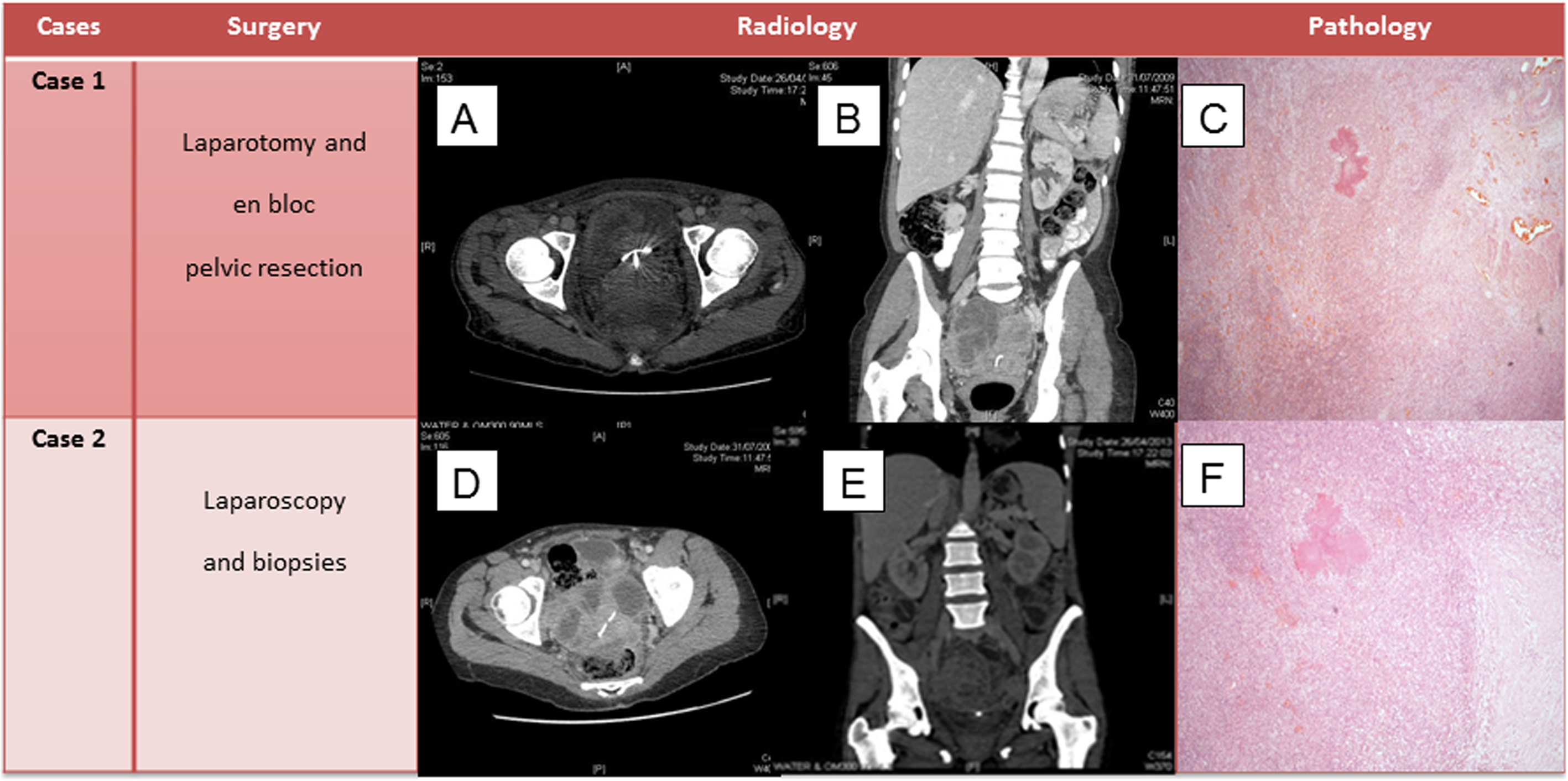

Supplement: Supplementary file 1 — Authors’ original file for figure 1 [file 40661_2014_5_MOESM1_ESM.tiff]

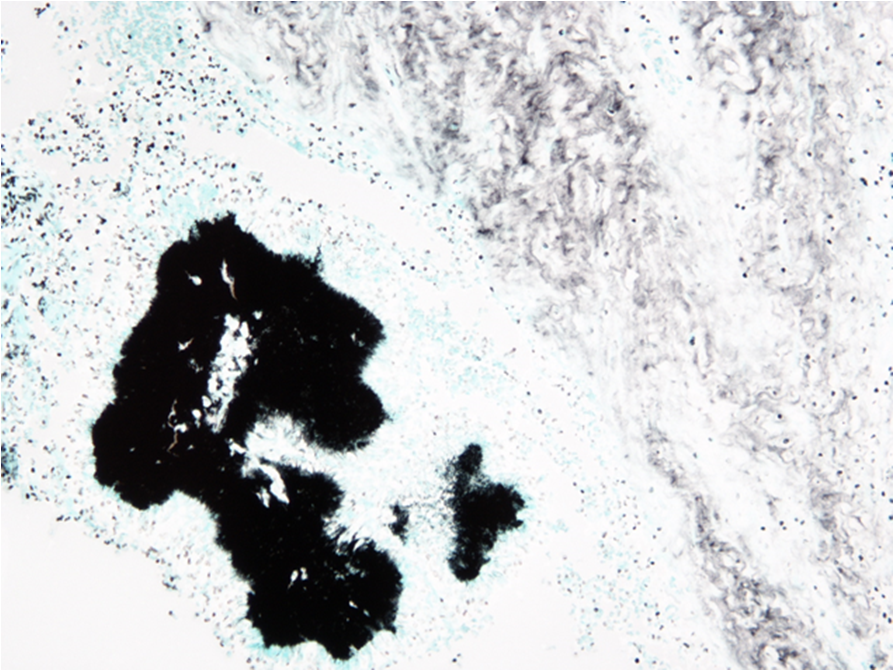

Supplement: Supplementary file 2 — Authors’ original file for figure 2 [file 40661_2014_5_MOESM2_ESM.tiff]

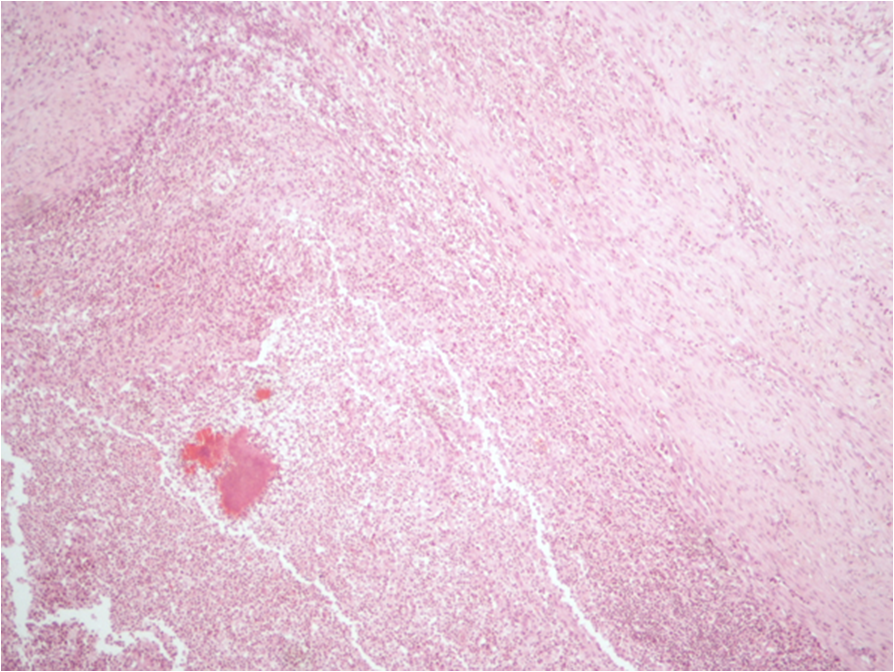

Supplement: Supplementary file 3 — Authors’ original file for figure 3 [file 40661_2014_5_MOESM3_ESM.tiff]

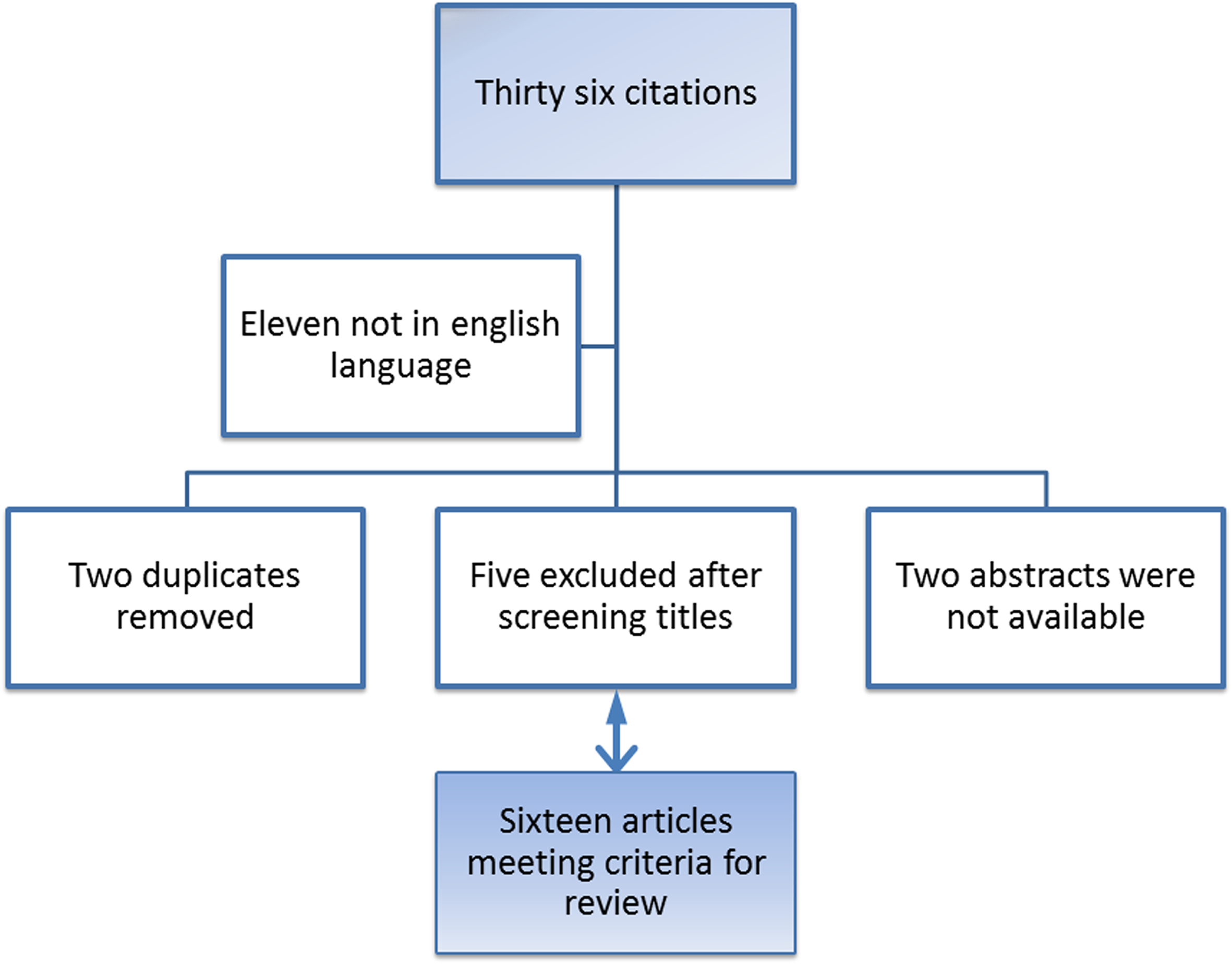

Supplement: Supplementary file 4 — Authors’ original file for figure 4 [file 40661_2014_5_MOESM4_ESM.tiff]

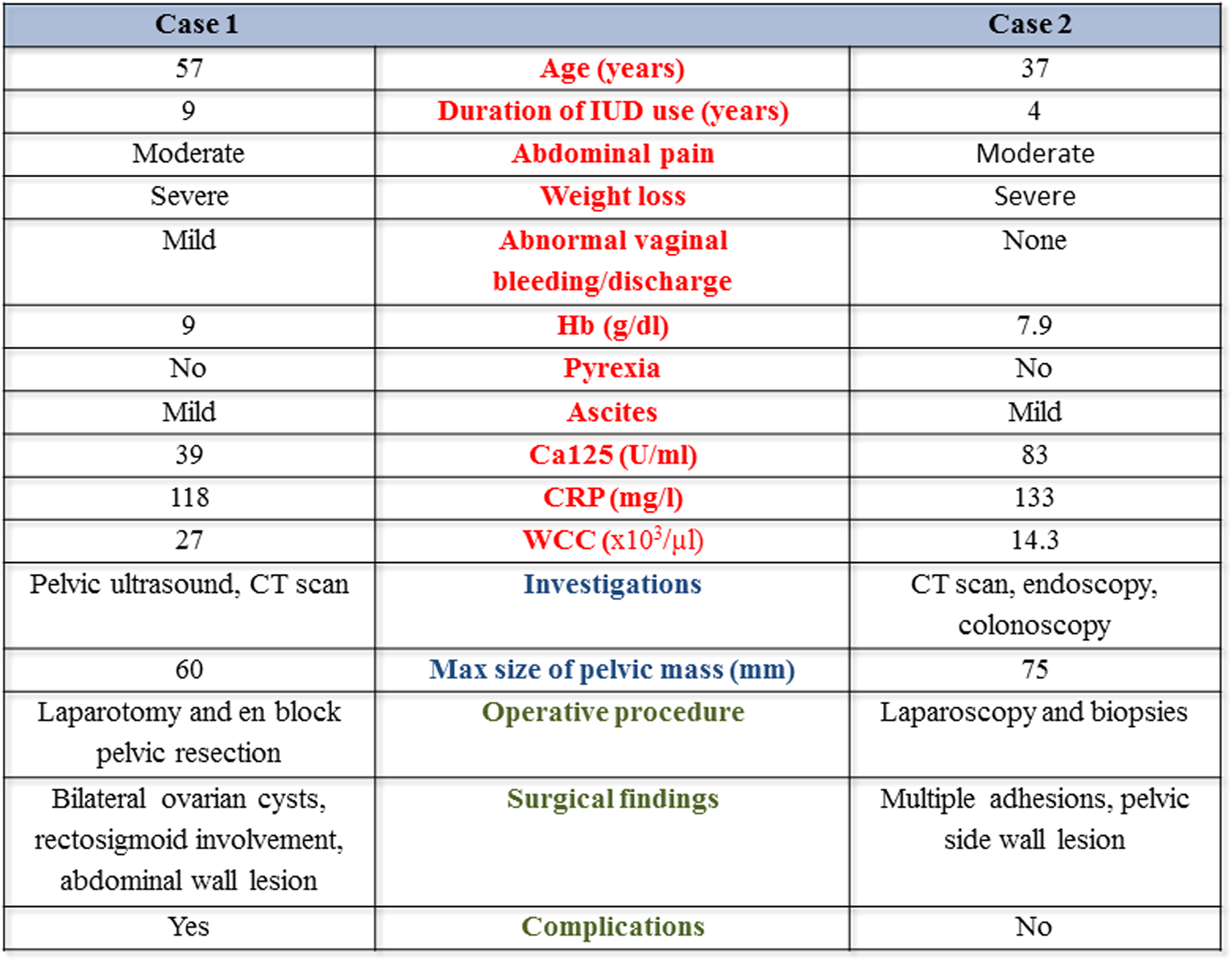

Supplement: Supplementary file 5 — Authors’ original file for figure 5 [file 40661_2014_5_MOESM5_ESM.tiff]
